# A Low-Cost Portable Machine Vision System for Non-Destructive Density Estimation of *Dunaliella* sp. Cultures

**DOI:** 10.3390/s26206487

**Published:** 2026-10-13

**Authors:** Jingjing Shan, Xinying Miao, Menghan Liu, Haoran Li, Zhipeng He, Zhenghan Wang, Han Wang

**Affiliations:** 1 College of Information Engineering, Dalian Ocean University, Dalian 116023, China; shanjingjing@dlou.edu.cn (J.S.); lmh0903@dlou.edu.cn (M.L.); 13842279398@163.com (H.L.); hezhipeng@dlou.edu.cn (Z.H.); 19883296810@163.com (Z.W.); 2 Liaoning Provincial Key Laboratory of Marine Information Technology, Dalian Ocean University, Dalian 116023, China; 3 College of Fisheries and Life Science, Dalian Ocean University, Dalian 116023, China; 18751872831@163.com

**Keywords:** *Dunaliella* sp., RGB imaging, microalgal density, non-destructive monitoring, controlled illumination, machine learning, edge computing, Raspberry Pi

## Abstract

**Highlights:**

**What are the main findings?**

- The system combines dark-box flask RGB imaging, mask-derived liquid-region extraction, and Raspberry Pi inference; KNN performed best ($R^{2}$ = 0.98, MAPE 6.41%).
- Each load-to-unload cycle took about 37 s (about 6 s processing); a 10-sample edge check showed density tracking ($R^{2}$ = 0.94).
- Leave-one-flask-out analysis supported transfer within the overlapping density range, but 41 out-of-range high-density cases were systematically underestimated.

**What are the implications of the main findings?**

- Once calibrated, the offline workflow enables non-destructive monitoring under fixed lab conditions, with periodic checks and range coverage.
- A recurrent color-based feature core appeared in both flasks; generalization to independent batches or new optical setups remains unestablished.

**Abstract:**

Routine microalgal cell counting requires sampling and microscopy. We developed a low-cost portable machine-vision system (LPMVS) for non-destructive density estimation of visibly green *Dunaliella* sp. cultures. The compact benchtop LPMVS combines motorized flask loading, a light-isolated dark box with diffuse light-emitting diode (LED) illumination, fixed camera–flask geometry, red–green–blue (RGB) imaging through the flask, mask-derived liquid-region extraction, Raspberry Pi inference, and a local liquid-crystal display (LCD). A ready-state load-to-unload cycle required approximately 37 s, including 6 s of processing. The two 40-day flask trajectories yielded 240 image–hemocytometer pairs; 200 records were used to compare three regressors with 20 features selected from 339 descriptors. K-nearest neighbors (KNN) performed best in the flask-mixed record-level comparison ($R^{2}$ = 0.98; mean absolute percentage error (MAPE) = 6.41%; n=40). Reciprocal leave-one-flask-out transfer analysis on complete trajectories with independently retrained pipelines gave KNN $R^{2}$ values of 0.914 and 0.803. In the reverse direction, $R^{2}$ was 0.936 within the overlapping range (n=79), but 41 above-range densities were systematically underestimated. Bootstrap resampling identified recurrent color-based features in both trajectories. An edge-device check using 10 additional measurements yielded preliminary reference-density tracking ($R^{2}$ = 0.94). These findings support the feasibility of the LPMVS workflow for non-destructive monitoring under controlled laboratory conditions, but extrapolation and generalization to independent batches and optical configurations remain unestablished.

## 1. Introduction

Microalgae are cultivated as sources of biomass and pigments for food, feed, and environmental applications [1,2,3,4,5]. *Dunaliella* sp. is a representative halotolerant species whose carotenoid accumulation and visible appearance vary with salinity, nutrient status, and light exposure [6,7,8,9]. Process-integrated sensing studies illustrate the broader shift toward automated monitoring [10,11]. For process monitoring, culture density is a primary measurable variable, although it does not alone define physiological state, growth phase, or harvest readiness.

Reference density is commonly obtained by hemocytometer or microscopic counting, dry-weight determination, optical density, fluorescence, or chemical analysis [12,13,14,15]. Hemocytometer counting requires sample removal and often dilution or fixation, chamber loading, microscopy, and trained observers; it is laborious and can interrupt cultivation. Optical and spectroscopic methods can support in-situ or online monitoring, but they often require specialized instrumentation and calibration for a defined biological and optical configuration [12,14,15]. These constraints motivate a low-cost, non-destructive red–green–blue (RGB) workflow that can repeatedly measure the culture through its flask without routine sampling.

Because salinity and nutrient stress can alter the visible color of *Dunaliella* sp. cultures, the present RGB calibration was restricted to visibly green cultures maintained under one controlled salinity condition [7,8,9].

Low-cost optical and image-based methods offer a route to faster and less destructive monitoring. Low-cost RGB sensing has been tested for online microalgae concentration measurement in photobioreactors [16]. Optical sensor systems have also been used to monitor and control biomass concentration automatically during cultivation [17]. Visible–near-infrared instruments have extended low-cost measurement to density and mass estimation [18], and a separate optical device has demonstrated real-time concentration measurement [19]. Image-based methods are attractive because the visible appearance of an algal suspension can serve as a low-cost measurement signal. Multivariate image analysis has been applied to microalgae monitoring in microscale photobioreactors [20]. Hue, saturation, and intensity analysis has been used for low-cost, non-destructive biomass quantification [21], while RGB image processing provides another non-invasive route under controlled imaging conditions [22].

More information-rich imaging has expanded the available monitoring strategies. Spectral images combined with convolutional neural networks can assess microalgal species, biomass, and spatial distribution [23]. Hyperspectral imaging has been reported for non-invasive cultivation monitoring [24], and near-infrared spectral imaging has been applied to lipid monitoring [25]. Quantum-dot light-emitting diodes combined with image edge detection have also been used for microalgal density assessment [26]. Current platforms include smartphone imaging [27], modular RGB frameworks for real-time neural inference [28], low-cost computer vision for cell-concentration estimation [29], and flow-imaging microscopy for monitoring and taxonomic analysis [30].

For practical image-based prediction, optical acquisition and regression calibration need to be designed together. Low-cost density monitoring has been studied with Gaussian-process modeling [31]. Least-squares and Gaussian-process models have also been compared for image-based microalgal density estimation [32]. Later studies incorporated the least absolute shrinkage and selection operator (LASSO) with image-texture features [33] and convolutional-neural-network regression [34], whereas deep-learning comparisons have focused on cell counting from microscope images [35]. These studies demonstrate the value of image-derived calibration, but they differ in biological materials, optical configurations, analyzed image regions, and deployment requirements. Suspension appearance can vary with pigment composition, optical scattering, container geometry, reflections, background regions, and reduced contrast at low or very high density. Moreover, the representative literature does not consistently evaluate automated flask handling, mask-derived whole-liquid-region analysis, embedded prediction, and system-level reporting of cost, size, mass, and runtime within one workflow. Table 1 therefore summarizes selected deployment-relevant characteristics on a qualitative, source-reported basis and positions the present system without claiming exhaustive coverage of all published platforms.

To provide an integrated and deployment-oriented sensing workflow, we developed a low-cost portable machine-vision system (LPMVS) for calibrated, non-destructive density estimation of *Dunaliella* sp. in transparent culture flasks. The LPMVS combines diffuse illumination, fixed camera–flask geometry, motorized flask loading, automatic light shielding, mask-derived liquid-region extraction, handcrafted image-feature analysis, lightweight regression, and Raspberry Pi-based local inference. Its contributions are fourfold: (1) a compact benchtop device for standardized RGB acquisition through transparent culture flasks; (2) non-destructive measurement after calibration without routine sample removal; (3) a machine-vision pipeline that reduces 339 handcrafted descriptors to a selected 20-feature subset and compares KNN, RF, and HGB regression models; and (4) local edge deployment with image acquisition, prediction, LCD display, and system-level evaluation of cost, dimensions, mass, model size, and processing time. The contribution is therefore integrative rather than algorithmic. The LPMVS was calibrated and evaluated for visibly green cultures prepared under one controlled seawater-based f/2 condition with a practical salinity of 35 ± 1. It was not designed to quantify salinity-dependent pigment production or cultures with stress-induced color changes, and validation beyond this controlled domain remains necessary.

The primary record-level analysis used 200 records selected from 240 sequential image–hemocytometer pairs obtained from two culture flasks. Forty early-stage records were excluded before model development because culture density changed slowly during the early cultivation period, resulting in many temporally adjacent observations within a narrow low-density range. The complete 240-record trajectories were retained for reciprocal leave-one-flask-out analysis, which was used to assess between-flask transfer over the full observed temporal trajectories. Thus, the two analyses were designed to address different questions: record-level internal comparison and flask-level transfer, respectively.

## 2. Materials and Methods

### 2.1. System Design, Portability, and Operation

**Figure 1:**
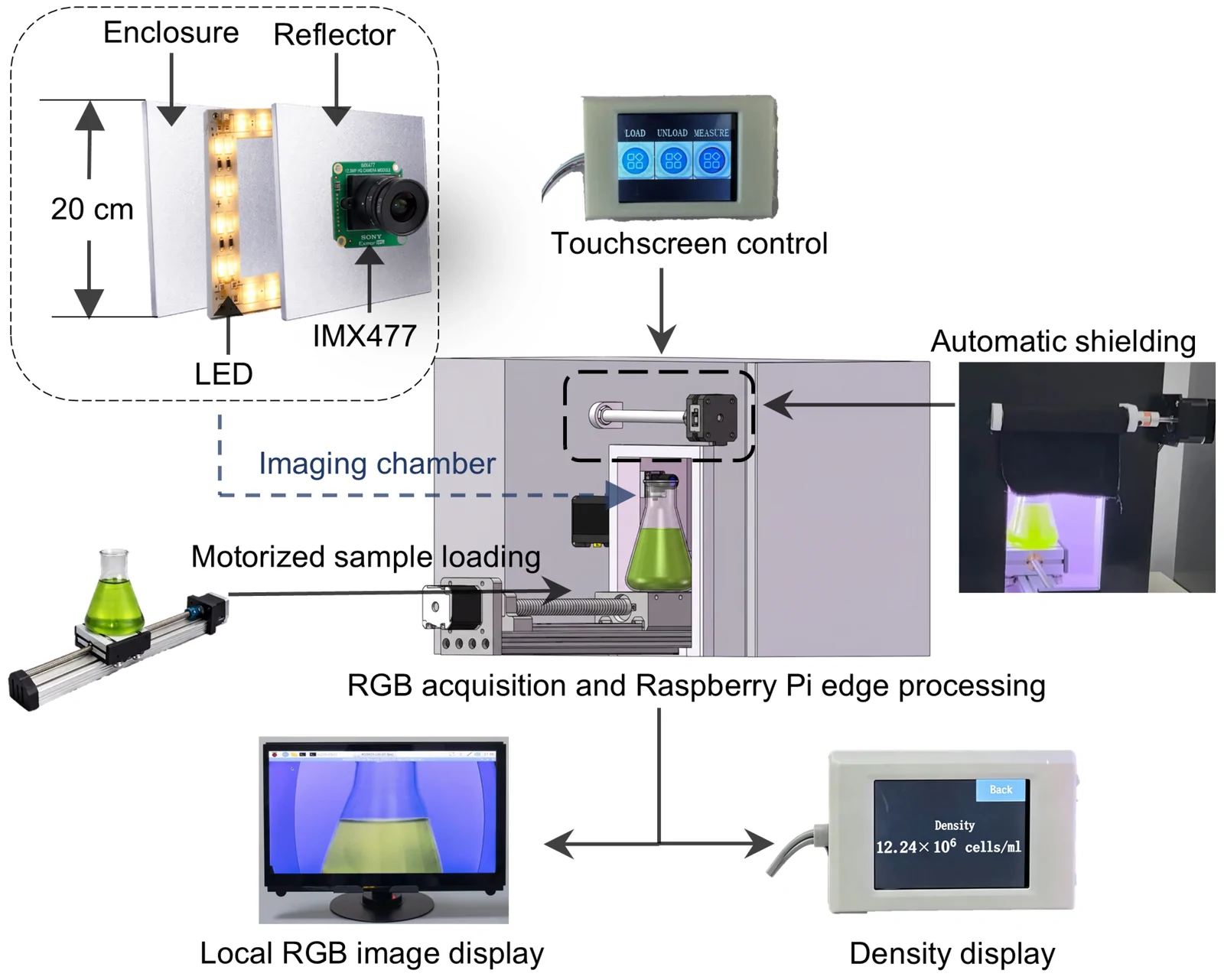
Hardware architecture and automated sensing workflow of the low-cost portable machine-vision system (LPMVS), including diffuse light-emitting-diode (LED) illumination, motorized flask loading, automatic light shielding, RGB image acquisition, Raspberry Pi edge processing, touchscreen control, and predicted-density display.

The LPMVS was designed to acquire repeatable RGB images directly through a transparent glass culture flask and to execute the calibrated prediction workflow locally. The device occupied an envelope within 30 × 30 × 20 cm, weighed less than 3 kg, and comprised six functional modules: motorized sample transport, a light-isolated imaging chamber, an RGB camera, a Raspberry Pi edge-computing unit, a touchscreen/LCD interface, and controlled internal illumination (Figure 1). Here, portable denotes a self-contained benchtop unit that can be moved between laboratory work areas and operated without an external computer.

A motor-driven linear rail transported the flask from the loading position to a fixed imaging position. A motorized light-shielding curtain closed after sample entry to isolate the chamber from ambient-light variation. An 18 W, 6000 K white light-emitting diode (LED) provided illumination, and a frosted reflector between the LED and the flask reduced glare and produced diffuse light. The camera and flask positions were mechanically fixed to maintain a consistent optical path, viewing angle, working distance, and analyzed image geometry across measurements. This configuration improved the comparability of image features by reducing variation in image acquisition geometry.

A Raspberry Pi High Quality Camera (Raspberry Pi Ltd, Cambridge, UK) equipped with a Sony IMX477 sensor (Sony Semiconductor Solutions Corporation, Atsugi, Japan) and a 6 mm focal-length lens acquired and stored JPEG images under the fixed acquisition configuration. The camera center was aligned with the liquid center, and the distance from the camera center to the central plane of the liquid was fixed at 13 cm. A Raspberry Pi 4 Model B (Raspberry Pi Ltd, Cambridge, UK) with 8 gigabytes (GB) of RAM and active cooling performed image storage and local processing.

For each measurement, the operator placed a flask on the rail and pressed the load button on the touchscreen. The rail moved the flask into the chamber, the curtain lowered, and the RGB image was acquired. After calibration, the Raspberry Pi segmented the liquid region, extracted the selected features, predicted density, and displayed the result on the LCD. Pressing the unload button raised the curtain and returned the flask.

The LPMVS was assembled from commercially available components. Its hardware cost was estimated from the purchase prices of the integrated components at the time of construction.

### 2.2. Culture Samples and Reference Measurements

The *Dunaliella* sp. culture was supplied by the National Marine Environmental Monitoring Center. Natural seawater was collected from the Heishijiao coastal area of Dalian, China. The seawater was filtered through a 0.45 μm membrane, sterilized at 121 °C for 20 min, and cooled to room temperature before use. The culture medium was then prepared according to the standard Guillard f/2 formulation [36,37] by adding the corresponding f/2 nutrient components to the treated seawater. No modification was made to the standard f/2 nutrient composition.

Two identical transparent culture flasks, each with a nominal capacity of 150 mL, were used. Each flask contained a working volume of 100 mL of algal culture. Cultures were maintained in an illuminated incubator at 25 ± 1 °C under a 12:12 h light/dark photoperiod, an incident light intensity of 100 μmol photons m^−2^ s^−1^, and a practical salinity of 35 ± 1. The calibration dataset was restricted to cultures that remained visibly green under these controlled conditions.

The two flasks, designated Flask A and Flask B, were inoculated independently and cultivated in parallel under the same environmental conditions. They represented two culture trajectories from one experimental preparation rather than 240 independent biological cultures or a replicated batch-level study. During the 40-day monitoring period, each flask was measured three times per day. For each measurement, one JPEG image and one corresponding hemocytometer reference measurement were obtained, yielding 120 image–reference pairs per flask and 240 pairs in total. Serial dilution was not used to create the imaged density range. Before each paired measurement, the flask was gently mixed so that cells were redistributed without vigorous bubble formation.

**Figure 2:**
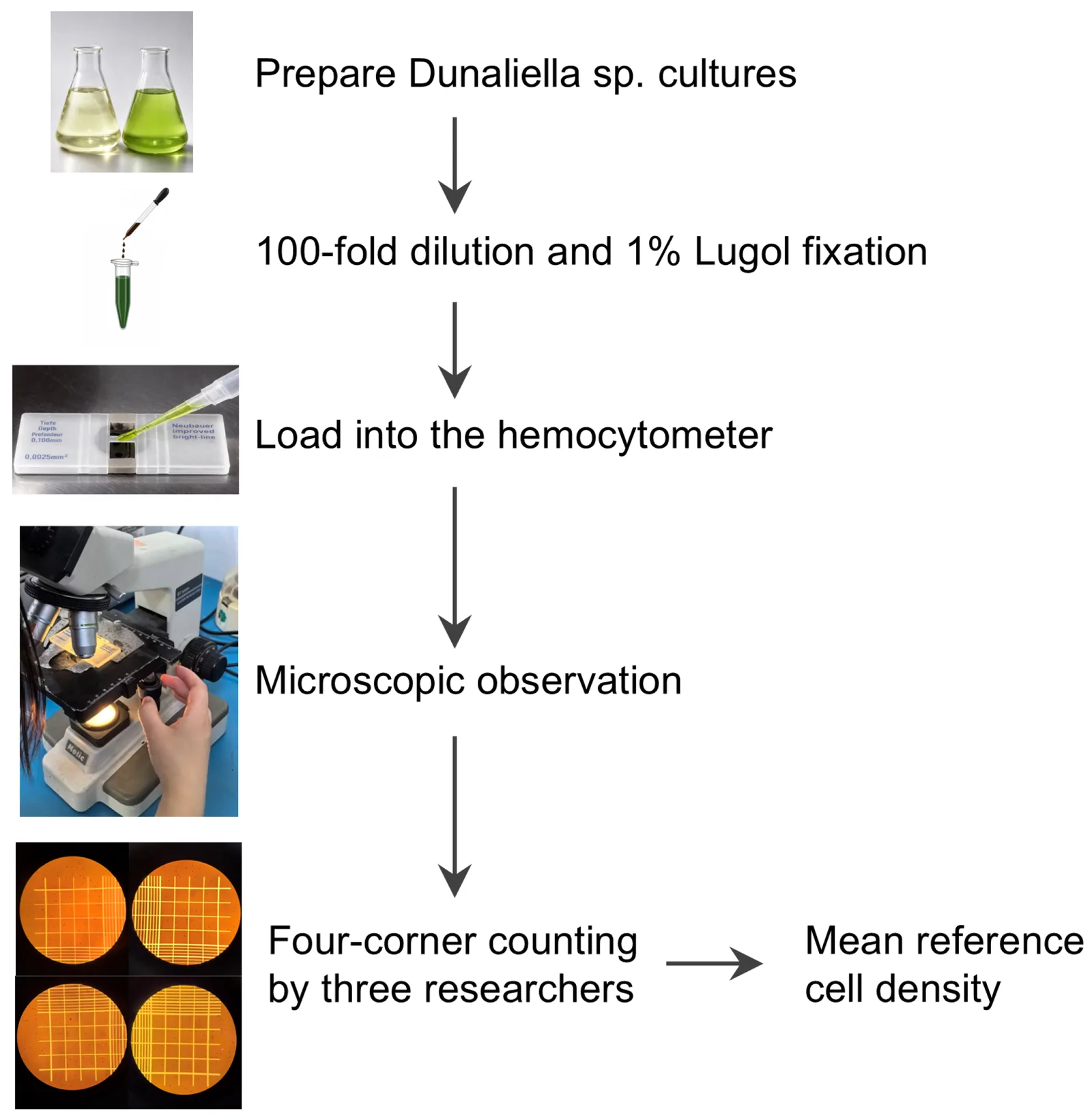
Hemocytometer-based workflow for obtaining reference density measurements. The procedure included 100-fold dilution, fixation with 1% Lugol’s iodine solution, hemocytometer loading, microscopic observation, counting of the four corner squares by three trained researchers, and calculation of the mean reference density.

For reference measurement, an aliquot was diluted 100-fold, fixed with 1% Lugol’s iodine solution, loaded into a standard Xiangbo hemocytometer (model 120604002; chamber depth, 0.1 mm), and observed at 100× magnification using a Motic biological microscope (Figure 2). The dilution and fixation procedures were applied only to the aliquot used for reference counting and did not alter the culture remaining in the flask imaged by the LPMVS. Cells in the four large corner squares were counted, and the original density was calculated using Equation (1):

(1) $$C=\left(\frac{N_{1}+N_{2}+N_{3}+N_{4}}{4}\right)\times 10^{4}\times D,$$

where *C* is the original density in cells mL^−1^, $N_{1}$–$N_{4}$ are the counts in the four 1 mm^2^ corner squares, D=100 is the dilution factor, and $10^{4}$ mL^−1^ is the chamber-volume conversion factor for a square with a depth of 0.1 mm. Cells touching the upper and left boundaries were included, whereas those touching the lower and right boundaries were excluded.

Three trained researchers independently counted each sample, and their mean was used as the reference density. A sample was recounted when the difference between any two observer counts exceeded five cells. All 240 candidate image–reference pairs satisfied the predefined reference-counting quality-control procedure.

During the early cultivation stage, cell density increased slowly, and many temporally adjacent observations were concentrated within a narrow low-density interval. To avoid overrepresenting this slowly changing early-stage condition in the primary record-level analysis, 20 early-stage records were excluded from each flask before model development according to a predefined temporal exclusion rule. The exclusion was applied symmetrically to the two flasks and was not based on image quality, feature values, model predictions, or prediction errors.

The resulting 200-record subset, consisting of 100 records from each flask, was used for the primary record-level model comparison. The complete 240-record trajectories were retained for the supplementary flask-specific bootstrap and reciprocal leave-one-flask-out analyses. Thus, the 200-record and 240-record datasets served different analytical purposes and were not treated as a single pooled performance estimate. The 200-record primary-analysis dataset covered 2.5 × $10^{6}$ to 100 × $10^{6}$ cells mL^−1^.

### 2.3. Image Segmentation and Feature Extraction

JPEG images acquired by the IMX477-based camera system under one fixed acquisition configuration were used throughout the analysis. The camera parameters were fixed at a sensor-sensitivity setting of 100, a shutter time of 13,000 μs, a red-channel white-balance gain of 2.10, and a blue-channel white-balance gain of 1.50. Automatic exposure, automatic white balance, and automatic gain control were disabled. Fixed acquisition settings, the dark-box chamber, and the unchanged camera–flask geometry reduced variation unrelated to density.

Image segmentation retained the effective liquid region and excluded most background, flask-wall, and reflection regions. Three high-density images with clear foreground-background contrast were used to generate the reference masks mask_A, mask_B, and mask_C. All three images used to construct the reference-mask library were selected from the training set and were not included in the internal comparison set. For every pixel, the five-dimensional vector in Equation (2) combined the perceptual CIE L*a*b* (CIELAB) color components, the saturation component of hue–saturation–value (HSV) color space, and the normalized Sobel edge-gradient magnitude from the grayscale image:

(2) $$x_{p}=\left[L_{p}^{*},\;a_{p}^{*},\;b_{p}^{*},\;S_{p},\;G_{\mathrm{Sobel},p}\right],$$

where $L_{p}^{*}$, $a_{p}^{*}$, and $b_{p}^{*}$ are the CIELAB components of pixel *p*, $S_{p}$ is its HSV saturation, and $G_{\mathrm{Sobel},p}$ is its normalized Sobel gradient magnitude.

For each high-density template, the pixel matrix was clustered using K-means with K=2 and random state = 42. The cluster occupying the central liquid region was converted to a binary mask. The three resulting masks were stored as mask_A, mask_B, and mask_C for subsequent template matching.

For each acquired image, a provisional foreground binary mask was generated by grayscale conversion, Gaussian blurring for denoising, and threshold segmentation. The intersection-over-union (IoU) between this provisional mask and each of mask_A, mask_B, and mask_C was calculated as the number of intersection pixels divided by the number of union pixels. The reference mask with the highest IoU was selected for each input image. In the present prototype, no experimentally validated absolute IoU rejection threshold was applied; therefore, the system did not automatically reject images with a low maximum IoU. Images acquired under substantially changed flask positions, fill volumes, illumination conditions, or camera geometry may produce an inappropriate liquid-region mask and require reference-paired verification or recalibration.

**Figure 3:**
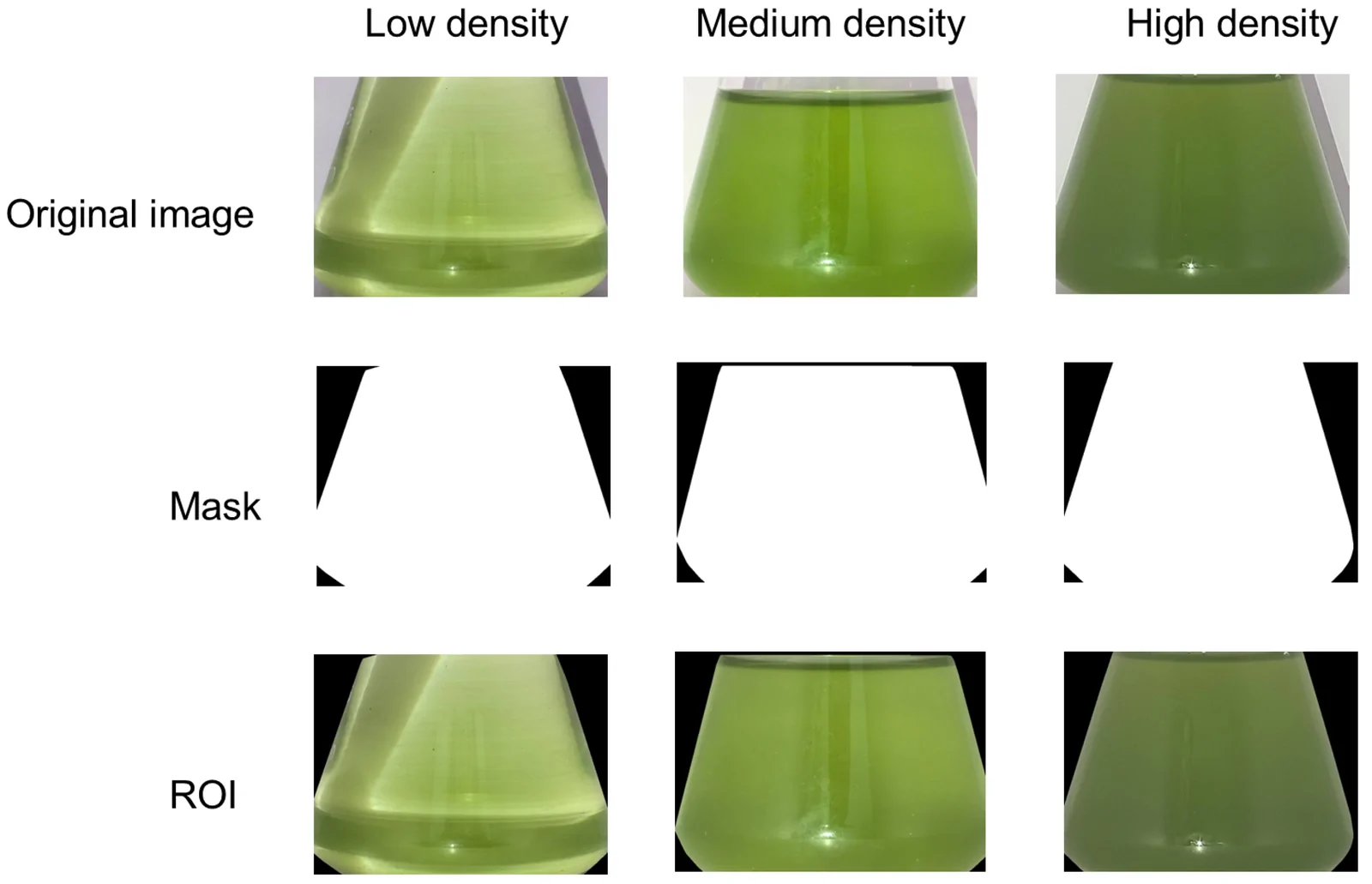
Representative RGB images and liquid-region extraction results for low-, medium-, and high-density visibly green *Dunaliella* sp. cultures. The rows show the original RGB image, the selected liquid-region mask, and the resulting effective liquid region of interest (ROI), respectively. Each displayed mask was selected from the three-mask template library generated from high-density images.

The selected mask was applied to remove the dark background, flask-wall regions, and major reflection regions. Image features were calculated only from the effective liquid region. Pixels outside the selected mask were set to black, the largest external contour defined the crop, and a 10-pixel padding margin was retained on all sides. The provisional threshold mask was used only for template matching. Adaptive ROI dimensions defined by the selected mask boundary were retained rather than forcing every crop to a fixed small window (Figure 3).

A total of 339 handcrafted features were extracted from each ROI. The feature pool included channel statistics and histogram proportions from RGB, HSV, CIELAB, YCbCr, and grayscale representations; vegetation and channel-ratio descriptors such as the visible atmospherically resistant index (*VARI*), excess green (*ExG*), excess red (*ExR*), normalized green–red difference index (*NGRDI*), color index of vegetation extraction (*CIVE*), and RGB ratios; and texture descriptors from gray-level co-occurrence matrices (GLCM) and local binary patterns (LBP). Color features represented changes in hue, brightness, and opacity, whereas texture features represented local spatial heterogeneity. In feature names, the prefix identifies the source channel (R, G, and B for RGB; H, S, and V for HSV; L, a, and b for CIELAB; Y, Cb, and Cr for YCbCr; and gray for grayscale), while the suffix denotes the statistic: q10, q25, q50, q75, and q90 are channel percentiles, median and mean are the channel median and mean, std is the standard deviation, iqr the interquartile range, skew the skewness, kurt the kurtosis, and hist the histogram-bin proportion. Image segmentation and feature extraction were implemented in Python 3.8.10 using NumPy 1.17.4 and OpenCV 4.2.0.

### 2.4. Feature Selection and Model Training

The 200 retained image–reference pairs were divided into a training set (n=160) and an internal comparison set (n=40) at a ratio of 8:2 using random state = 42. The split was performed at the measurement-record level rather than at the flask level. Consequently, records from the same flask could occur in both subsets, and the split was flask-mixed. The resulting internal comparison therefore characterized record-level consistency under the tested acquisition conditions rather than generalization to an independently cultivated culture. Density was binned at 10 × $10^{6}$ cells mL^−1^ intervals for stratified sampling. Low, medium, and high density were defined as $C<30\times 10^{6}$, $30\times 10^{6}\leq C<60\times 10^{6}$, and $C\geq 60\times 10^{6}$ cells mL^−1^, respectively. All feature filtering, scaling, supervised feature selection, and hyperparameter optimization steps were fitted using only the 160-record training set. The resulting transformations and fixed top-20 feature subset were then applied to the 40-record internal comparison set without refitting.

Redundancy filtering first ranked the 339 features by mutual information with density and sequentially retained a feature only when its absolute Pearson correlation with all previously retained features was below 0.97. This reduced the feature count from 339 to 158. Min–max scaling was then fitted on the training set for low-variance filtering; features with scaled variance ≤0.03 were removed, leaving 122 candidates.

The remaining candidates were ranked by absolute Pearson correlation, absolute Spearman correlation, and mutual information. For each feature, the three ranking positions were averaged using Equation (3):

(3) $${\mathrm{MeanRank}}_{i}=\frac{R_{i}\left(P\right)+R_{i}\left(S\right)+R_{i}\left(\mathrm{MI}\right)}{3},$$

where $R_{i}\left(P\right)$, $R_{i}\left(S\right)$, and $R_{i}\left(\mathrm{MI}\right)$ are the ranking positions of feature *i* under the three relevance criteria. A rank-based importance score was then calculated using Equation (4):

(4) $${\mathrm{Score}}_{i}=K-{\mathrm{MeanRank}}_{i},$$

where K=93.333 is one plus the largest mean rank among the selected 20 features. This score was used only to order the retained descriptors in the reported feature list; feature selection itself was determined by ascending mean rank. The 20 features with the best fused ranks were retained as fixed model inputs.

Three regression algorithms were compared: k-nearest neighbors (KNN), random forest (RF) [38], and histogram-based gradient boosting (HGB). KNN represented a distance-based local model, RF a bagging-based tree ensemble, and HGB a boosting-based nonlinear model. The KNN prediction was calculated from the neighboring training observations using Equation (5):

(5) $$\hat{y}\left(x\right)=\frac{\sum_{i\in N_{k}\left(x\right)}w_{i}y_{i}}{\sum_{i\in N_{k}\left(x\right)}w_{i}},$$

where $N_{k}\left(x\right)$ is the set of *k* nearest neighbors of sample *x*, $y_{i}$ is the *i*th neighbor density, and $w_{i}$ is its weight. Median imputation and model-specific scaling were included in the KNN pipeline. RF was optimized with RandomizedSearchCV (60 iterations), and KNN and HGB were optimized with GridSearchCV. All searches used 10-fold cross-validation within the training set. Within each cross-validation fold, imputation and model-specific scaling were fitted only on the corresponding training subset; validation-fold and comparison-set observations were not used to estimate preprocessing parameters. The models were implemented in Python 3.8.10 using NumPy 1.17.4, pandas 0.25.3, scikit-learn 0.22.2.post1, joblib 1.4.2, and OpenCV 4.2.0 [39]. Table 2 reports the selected hyperparameters.

The KNN grid search evaluated StandardScaler and RobustScaler, odd neighbor values from 3 to 21, uniform and distance weighting, p=1 and p=2, and leaf sizes of 20, 30, and 40. The final setting of 17 neighbors with uniform weighting was selected because it produced the best cross-validated training-set score among the prespecified alternatives. The RF randomized search (60 iterations) used n_estimators of 200, 400, 600, 800, 1000, and 1200; max_depth of None, 4, 6, 8, 10, 12, and 16; min_samples_split of 2, 4, 6, 8, and 10; min_samples_leaf of 1–4; max_features of sqrt, log2, 0.3, 0.5, 0.7, and 1.0; and bootstrap values of True and False. The HGB grid used learning_rate values of 0.02, 0.03, and 0.05; max_iter of 300 and 500; max_depth of 5, 6, and 8; max_leaf_nodes of 31 and 63; min_samples_leaf of 10 and 20; and l2_regularization of 0.0 and 0.1.

### 2.5. Bootstrap Feature Stability Analysis

To evaluate the stability of the feature-selection procedure, a supplementary bootstrap analysis was performed separately for Flask A and Flask B using the complete 120-record trajectory of each flask as its sampling pool. For each flask, 1000 bootstrap samples were generated by sampling its measurement records with replacement. Within each bootstrap sample, the complete feature-selection sequence was repeated, including redundancy filtering, low-variance filtering, and fused ranking based on Pearson correlation, Spearman correlation, and mutual information. The 20 highest-ranked features were retained in each iteration. Feature stability was summarized by the selection frequency of each descriptor, defined as the percentage of bootstrap iterations in which a descriptor appeared in the top-20 set, together with the distribution of its ranking position across bootstrap iterations. This analysis was used to assess the resampling stability of the selected descriptors and was not used to redefine the feature inputs of the primary model comparison. The two flask datasets were collected as continuous temporal trajectories, and records within each trajectory may therefore exhibit temporal autocorrelation. The stability analysis describes feature recurrence within the two observed culture trajectories and does not establish feature-selection stability across independently cultivated batches.

### 2.6. Model Evaluation and System-Level Deployment Assessment

The internal comparison set was reserved for a single comparison of the three prespecified optimized candidate pipelines after model tuning. It was not used during feature filtering, supervised feature selection, preprocessing-parameter estimation, or hyperparameter optimization. Performance was quantified using the coefficient of determination ($R^{2}$), root mean square error (RMSE), mean absolute error (MAE), mean absolute percentage error (MAPE), and bias using Equations (6)–(10). Here, $R^{2}$ describes how well predictions track variation in the reference values, RMSE gives greater weight to larger errors, MAE represents the typical absolute deviation, MAPE is the mean absolute relative error expressed as a percentage of reference density, and bias indicates systematic over- or underprediction. Nonparametric percentile bootstrap 95% confidence intervals were calculated from 5000 bootstrap resamples of the 40 comparison-set pairs, sampled with replacement using a fixed random seed of 42.

(6) $$R^{2}=1-\frac{\sum_{i=1}^{n}{\left(y_{i}-{\hat{y}}_{i}\right)}^{2}}{\sum_{i=1}^{n}{\left(y_{i}-\bar{y}\right)}^{2}},$$

(7) $$\mathrm{RMSE}=\sqrt{\frac{1}{n}\sum_{i=1}^{n}{\left(y_{i}-{\hat{y}}_{i}\right)}^{2}},$$

(8) $$\mathrm{MAE}=\frac{1}{n}\sum_{i=1}^{n}\left|y_{i}-{\hat{y}}_{i}\right|,$$

(9) $$\mathrm{MAPE}=\frac{100\%}{n}\sum_{i=1}^{n}\left|\frac{y_{i}-{\hat{y}}_{i}}{y_{i}}\right|,$$

where $y_{i}$ and ${\hat{y}}_{i}$ are the reference and predicted densities, $\bar{y}$ is the mean reference density, and *n* is the sample count. RMSE and MAE were reported in $10^{6}$ cells mL^−1^. All reference densities were positive (minimum, 2.5 × $10^{6}$ cells mL^−1^), so MAPE contained no zero-valued denominators. Prediction error $e_{i}$ and mean bias $\bar{e}$ were calculated using Equation (10), and the Bland–Altman 95% limits of agreement (LoA) were calculated using Equation (11):

(10) $$e_{i}={\hat{y}}_{i}-y_{i},\;\bar{e}=\frac{1}{n}\sum_{i=1}^{n}e_{i},$$

(11) $${\mathrm{LoA}}_{95\%}=\bar{e}\pm 1.96\;SD_{e}.$$

where $e_{i}$ is the difference for sample *i*, $\bar{e}$ is the mean difference (bias), $SD_{e}$ is the sample standard deviation of the differences, and LoA denotes the 95% limits of agreement.

After comparison on the internal comparison set, the selected KNN model underwent supplementary 10-fold cross-validation on the full 200-record primary-analysis dataset using the fixed top-20 feature set. Feature selection and reference-mask construction were not repeated within each fold. This analysis was therefore interpreted as a descriptive record-level stability check conditional on the fixed image-processing and feature-selection choices, rather than as fully nested validation.

To provide a stricter assessment of between-flask transfer over the complete observed trajectories, reciprocal leave-one-flask-out (LOFO) analysis was performed independently in both directions. The LOFO analysis used all 120 records from the training flask and all 120 records from the held-out flask, including the early-stage records excluded from the primary 200-record analysis. For each direction, reference-mask construction, feature filtering, feature selection, preprocessing, scaling, hyperparameter optimization, and model fitting were performed using the training flask only. The resulting pipeline was then applied to the held-out flask without refitting. No image, reference density, feature-selection result, preprocessing parameter, hyperparameter, or model-fitting information from the held-out flask was used during model development. The same three regression algorithms were retained, and performance was summarized using $R^{2}$, RMSE, MAE, and bias as defined for the primary comparison.

Because the density ranges of the two trajectories were not identical, LOFO performance was evaluated over both the full held-out trajectory and the density range shared with the training flask. For the Flask-B-trained/Flask-A-tested direction, the maximum density represented in the Flask B training trajectory was 65.25 × $10^{6}$ cells mL^−1^. Flask A observations above this value were therefore treated as extrapolation beyond the training-density range. For the selected KNN model, calibration and Bland–Altman analyses were additionally examined within the common calibration range (≤65.25 × $10^{6}$ cells mL^−1^) to distinguish between-flask transfer within the supported range from extrapolation behavior.

#### 2.6.1. Edge-Device Check

The selected KNN pipeline was saved as a .joblib file and loaded on the Raspberry Pi before timing. The deployment environment used Python 3.8.10, NumPy 1.17.4, pandas 0.25.3, scikit-learn 0.22.2.post1, joblib 1.4.2, and OpenCV 4.2.0. After system boot and model preload, flask loading and shield closure required approximately 13 s, image acquisition 5 s, the complete on-device model-processing interval 6 s, and shield opening and flask unloading 13 s. These stages yielded an approximate ready-state load-to-unload cycle of 37 s. A separate full-session measurement from power-on through one complete device test and shutdown required approximately 5 min.

An additional set of 10 edge-device measurements, separate from the 240-record two-flask trajectory dataset and therefore not included in the 200-record model-development subset, was used for the deployment check. These measurements were not used for reference-mask construction, feature selection, preprocessing, hyperparameter optimization, or model fitting. Their reference densities ranged from 20 to 80 × $10^{6}$ cells mL^−1^, and the saved pipeline was applied without refitting. This assessment was treated as a preliminary edge-device feasibility check rather than independent external validation.

#### 2.6.2. Cost Estimation

The prototype cost was estimated by summing the purchase prices of the integrated hardware components at the time of assembly. The estimate included the Raspberry Pi High Quality Camera (Raspberry Pi Ltd, Cambridge, UK), Raspberry Pi, LED light source, stepper motors, linear guide rail, polyvinyl chloride (PVC) enclosure, display, and other electronic and assembly components. It represented an approximate hardware acquisition cost rather than a commercial sale price. The estimate excluded the microscope, hemocytometer, culture medium, computer used for model development, and general laboratory infrastructure.

#### 2.6.3. Short-Term Instrument Repeatability

Short-term instrument repeatability was assessed separately from prediction accuracy using an undisturbed culture flask with a hemocytometer reference density of approximately 45 × $10^{6}$ cells mL^−1^. After the flask was positioned at the fixed imaging location, 10 consecutive RGB images were acquired without unloading or repositioning the flask and without changing the camera, illumination, or acquisition settings. The complete image-processing and KNN-inference pipeline was independently applied to each image.

Repeatability was summarized using the mean predicted density, standard deviation (SD), and coefficient of variation (CV), calculated as the standard deviation divided by the mean prediction and expressed as a percentage. This experiment characterized short-term repeatability of continuous image acquisition and inference under a fixed configuration. It did not evaluate variability caused by flask unloading, mechanical transport, repositioning, flask replacement, or changes in optical conditions.

## 3. Results

### 3.1. Feature Selection and Stability Analysis

The primary record-level analysis used the 200-record subset obtained after the predefined exclusion of 40 early-stage records from the complete 240-record dataset. The complete 120-record trajectory from each flask was used for the supplementary flask-specific bootstrap and reciprocal leave-one-flask-out (LOFO) analyses. Flask A covered 2.5–100 × $10^{6}$ cells mL^−1^, whereas Flask B covered 2.5–65.25 × $10^{6}$ cells mL^−1^ (Table 3). Therefore, 41 Flask A observations exceeded the maximum density represented in the Flask B trajectory. This density-range difference was considered when interpreting the LOFO results. In the 200-record primary-analysis subset, the 90–100 × $10^{6}$ cells mL^−1^ interval contained only two training observations and no internal comparison observations, indicating sparse coverage of the highest observed densities.

Feature processing reduced the original 339 descriptors to 158 after redundancy filtering and to 122 after low-variance filtering. Fused Pearson, Spearman, and mutual-information ranking then selected 20 features. The final top-20 feature set, listed in descending fused-ranking score, was *VARI*, *H_q25*, *R_q75*, *H_q10*, *H_q75*, *G_iqr*, *gray_skew*, *V_median*, *Y_hist_3*, *L_hist_5*, *b_std*, *H_mean*, *gray_hist_5*, *glcm_correlation_std*, *B_q75*, *lbp_16_2_6*, *R_kurt*, *B_skew*, *L_hist_6*, and *R_hist_3*. Color-related descriptors constituted most of this feature set under the tested imaging and cultivation conditions. *VARI* ranked first, followed by hue and red-channel statistics, including *H_q25*, *R_q75*, *H_q10*, and *H_q75*. GLCM correlation and an LBP descriptor were also retained but ranked below most color-related features.

These single-partition ranks describe feature relevance in the original training subset but do not by themselves establish selection stability; this issue was therefore examined using supplementary bootstrap resampling.

**Figure 4:**
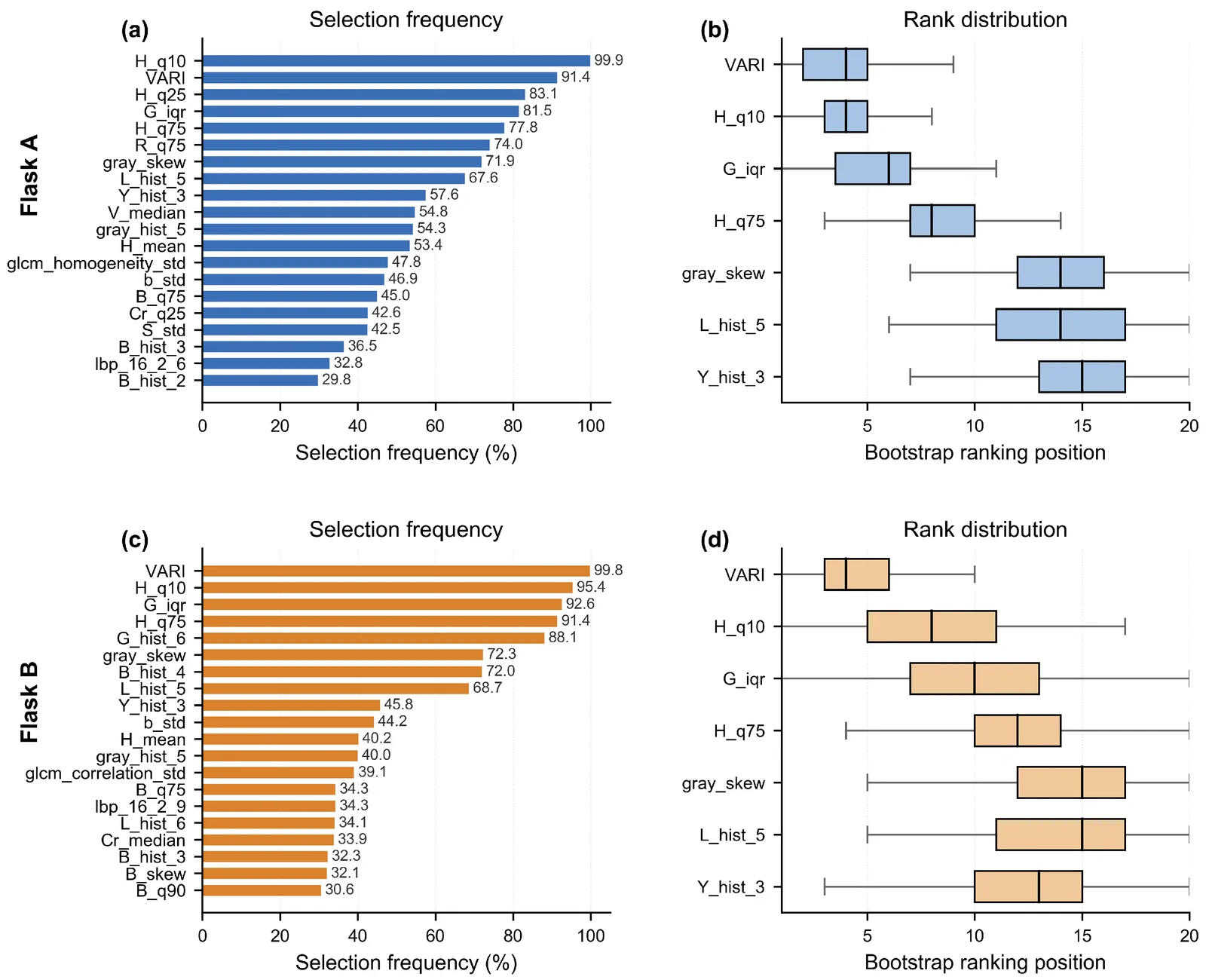
Bootstrap feature-selection stability for Flask A and Flask B. Panels (**a**,**c**) show the selection frequencies of the 20 descriptors most frequently retained in the bootstrap top-20 sets for Flask A and Flask B, respectively. Panels (**b**,**d**) show the bootstrap distributions of ranking positions for representative recurrent descriptors. Selection frequency denotes the percentage of 1000 bootstrap iterations in which a descriptor appeared in the top-20 set. The results describe resampling stability within the two observed culture trajectories and do not establish feature-selection stability across independent cultivation batches.

Bootstrap resampling identified recurrent descriptors within both observed culture trajectories (Figure 4). In Flask A, *H_q10* and *VARI* appeared in 99.9% and 91.4% of the bootstrap top-20 sets, respectively, followed by *H_q25* (83.1%), *G_iqr* (81.5%), *H_q75* (77.8%), and *gray_skew* (71.9%). In Flask B, *VARI* and *H_q10* were selected in 99.8% and 95.4% of the iterations, respectively, while *G_iqr* (92.6%), *H_q75* (91.4%), *G_hist_6* (88.1%), and *gray_skew* (72.3%) were also frequently retained. Several descriptors, particularly *VARI*, *H_q10*, *G_iqr*, *H_q75*, *gray_skew*, *L_hist_5*, and *Y_hist_3*, recurred in both flask-specific analyses.

The bootstrap ranking distributions showed that the most frequently selected descriptors generally remained near the top of the fused ranking, whereas lower-frequency descriptors exhibited broader and less consistent ranking positions. The recurrent features were dominated by color, hue, intensity, and channel-distribution information. Texture-related descriptors derived from GLCM or LBP were retained in some bootstrap samples but showed lower and less consistent selection frequencies. These results support the recurrence of a color-dominated feature core within the two observed culture trajectories. They do not establish feature-selection stability across independently cultivated batches, salinity conditions, stress states, or separately assembled imaging devices.

### 3.2. Regression Model Comparison and Record-Level Internal Validation

All three optimized regressors captured the positive association between image-derived features and reference density on the record-level internal comparison set (Table 4). KNN achieved the highest $R^{2}$ (0.98) and the lowest RMSE (2.84 × $10^{6}$ cells mL^−1^), MAE (2.16 × $10^{6}$ cells mL^−1^), and MAPE (6.41%). Its mean bias was −0.42 × $10^{6}$ cells mL^−1^, indicating limited average systematic deviation within this comparison set. RF also showed a strong reference–prediction association but produced larger absolute and relative errors. HGB showed the largest errors and the most negative mean bias among the three models. On the basis of these record-level results, KNN was selected for the subsequent edge-device assessment and detailed agreement analysis.

**Figure 5:**
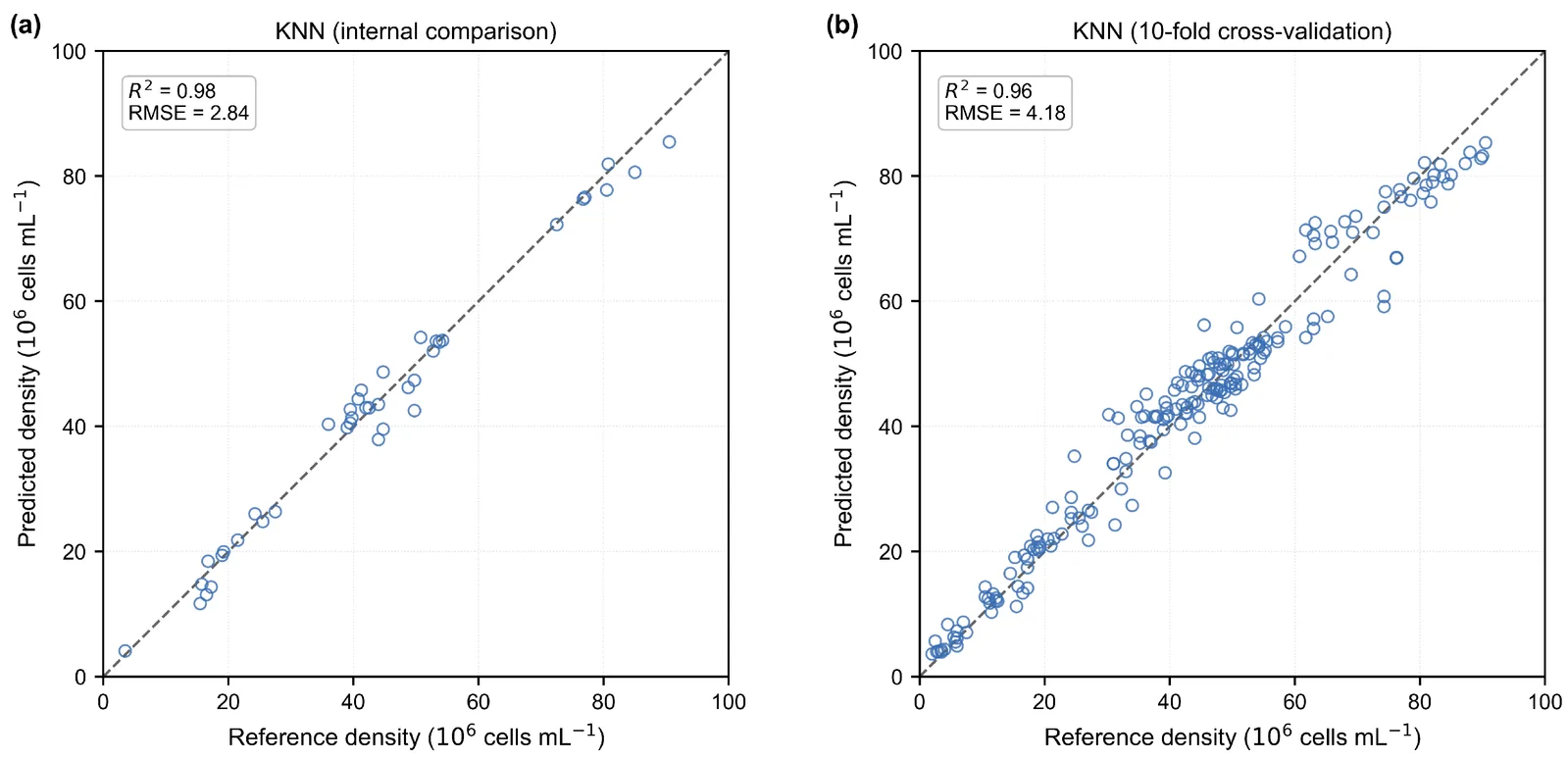
Record-level prediction performance of the selected k-nearest neighbors (KNN) model. (**a**) Performance on the 40-record internal comparison set obtained from the 8:2 measurement-record split. (**b**) Supplementary 10-fold cross-validation on the 200-record primary-analysis dataset using the fixed top-20 feature set. The dashed line represents the 1:1 relationship between reference and predicted density. Because the cross-validation analysis did not hold out an entire flask or reconstruct the reference-mask library and feature-selection procedure within each fold, it represents a conditional record-level stability check rather than independent-culture validation.

Figure 5a shows the KNN predictions for the 40-record internal comparison set. The observations were distributed close to the 1:1 line, consistent with the performance estimates in Table 4. Figure 5b presents a supplementary 10-fold cross-validation analysis of KNN on the 200-record primary-analysis dataset using the fixed top-20 feature set. This analysis yielded $R^{2}$ = 0.96 and RMSE = 4.18 × $10^{6}$ cells mL^−1^. The lower $R^{2}$ and higher RMSE relative to the single internal comparison split indicate variation across repeated record-level partitions. However, this analysis did not hold out an entire culture flask, and the reference-mask library and feature-selection procedure were not reconstructed within each fold. It should therefore be interpreted as a conditional record-level stability check rather than as evidence of transfer to an independently cultivated culture. Between-flask transfer was assessed separately using reciprocal leave-one-flask-out analysis in Section 3.3.

### 3.3. Reciprocal Leave-One-Flask-Out Transfer Analysis

Reciprocal leave-one-flask-out (LOFO) analysis was performed using the complete 120-record trajectory from each flask. The two transfer directions were trained and evaluated independently. In the Flask A → B direction, all model-development operations were performed using Flask A only, and the resulting pipeline was applied to Flask B without refitting. The same complete procedure was independently repeated in the Flask B → A direction. Reference-mask construction, feature filtering, feature selection, preprocessing, scaling, hyperparameter optimization, and model fitting were performed using the training flask only. No image, reference density, feature-selection result, preprocessing parameter, hyperparameter, or model-fitting information from the held-out flask was used during model development.

When Flask A was used for model development and Flask B was held out, all three regressors followed the general reference–prediction trend across the Flask B density range. KNN gave the strongest performance in this direction, with $R^{2}$ = 0.914, RMSE = 4.109 × $10^{6}$ cells mL^−1^, and MAE = 3.470 × $10^{6}$ cells mL^−1^ (Table 5). This result indicates useful transfer between the two observed culture trajectories under the tested density range, but it does not establish generalization to independent cultivation batches.

The reverse Flask B → A direction showed weaker full-range transfer. KNN achieved $R^{2}$ = 0.803, RMSE = 13.453 × $10^{6}$ cells mL^−1^, and MAE = 9.384 × $10^{6}$ cells mL^−1^ over the complete Flask A trajectory. The performance degradation was concentrated in the 41 Flask A observations above 65.25 × $10^{6}$ cells mL^−1^, which exceeded the maximum density represented in the Flask B training trajectory. In this high-density region, all three regressors showed systematic underestimation and a compressed prediction response. The similar degradation observed for KNN, RF, and HGB suggests that the observed error pattern was primarily associated with extrapolation beyond the training-density range rather than with the choice of a particular regression algorithm. However, differences between the two culture trajectories may also have contributed to the asymmetric transfer behavior.

When the Flask B → A analysis was restricted to the overlapping density range of ≤$65.25\times 10^{6}$ cells mL^−1^, KNN performance improved to $R^{2}$ = 0.936, RMSE = 4.669 × $10^{6}$ cells mL^−1^, and MAE = 4.095 × $10^{6}$ cells mL^−1^. This improvement indicates conditional between-flask transfer within the overlapping calibration domain, whereas the full-range result reflects both interpolation within the shared range and extrapolation above the training-flask boundary. The remaining 41 Flask A observations exceeded the maximum density represented in the Flask B training data and therefore constituted extrapolation rather than interpolation within the supported training range. All three models showed systematic underestimation in these observations, which contributed to the negative full-range biases in the Flask B → A direction (Table 5). The present models should not be used to infer densities beyond the range represented in the training trajectory without additional calibration data.

**Figure 6:**
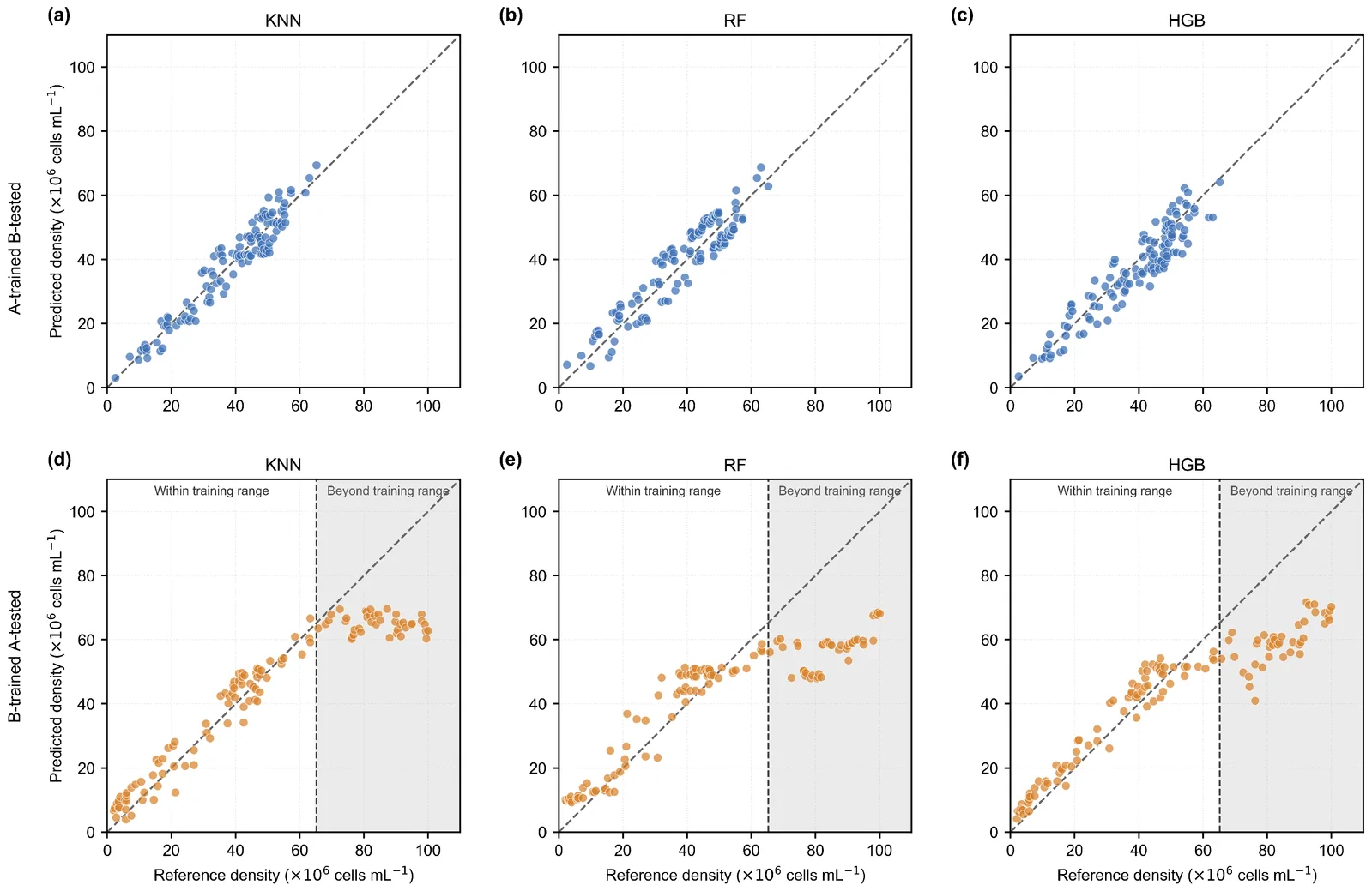
Reciprocal leave-one-flask-out transfer analysis for the three regression models. Panels (**a**–**c**) show models trained on Flask A and evaluated on the completely held-out Flask B trajectory; panels (**d**–**f**) show models trained on Flask B and evaluated on the completely held-out Flask A trajectory. The dashed diagonal line represents the 1:1 relationship between reference and predicted density. In panels (**d**–**f**), the vertical dashed line at 65.25 × $10^{6}$ cells mL^−1^ indicates the maximum density represented in the Flask B training trajectory. The shaded region denotes extrapolation beyond the training density range, rather than a separate experimental condition.

The reciprocal LOFO prediction patterns are illustrated in Figure 6.

**Figure 7:**
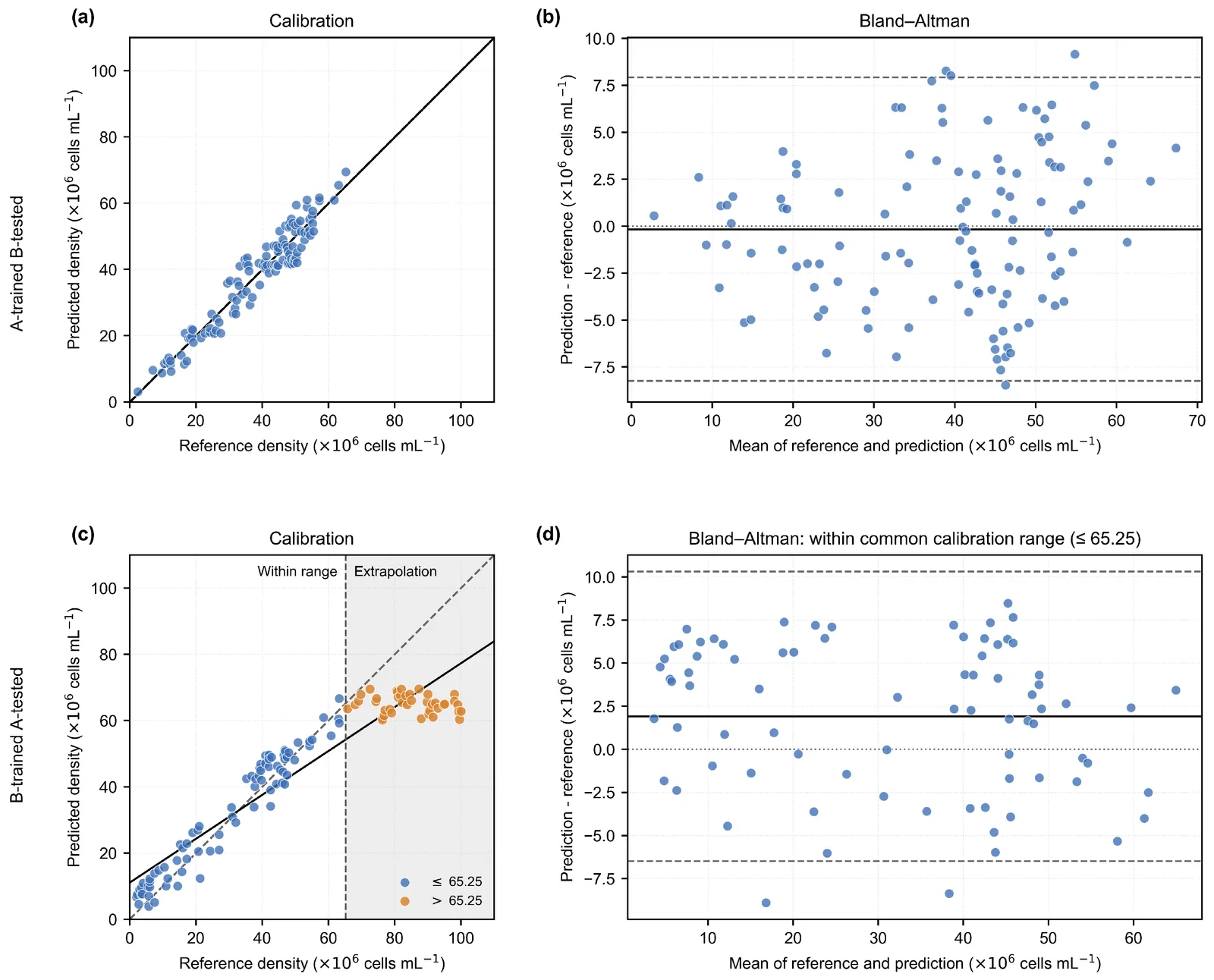
Reference–prediction agreement and Bland–Altman analysis for KNN under reciprocal leave-one-flask-out evaluation. (**a**) Flask-A-trained/Flask-B-tested prediction relationship and (**b**) corresponding Bland–Altman analysis. (**c**) Flask-B-trained/Flask-A-tested prediction relationship; observations above 65.25 × $10^{6}$ cells mL^−1^ are outside the Flask B training range and are shown as extrapolation. (**d**) Bland–Altman analysis for the Flask-B-trained/Flask-A-tested direction restricted to the common calibration range (≤65.25 × $10^{6}$ cells mL^−1^; n=79). In panels (**a**,**c**), diagonal lines indicate the 1:1 relationship; the solid line in panel (**c**) is the least-squares fit over all Flask-A observations. In panels (**b**,**d**), solid and dashed horizontal lines indicate mean bias and 95% limits of agreement, respectively, and the dotted horizontal line marks zero difference.

Because KNN showed the best overall LOFO performance, its calibration and agreement behavior was examined in greater detail. In the Flask-A-trained/Flask-B-tested direction, prediction differences remained comparatively balanced across the evaluated density range. In the reverse direction, systematic underestimation became evident once Flask A densities exceeded the maximum density represented in the Flask B training data. Restricting the agreement analysis to the common calibration range removed this high-density extrapolation segment and allowed between-flask agreement within the supported density domain to be assessed separately (Figure 7).

Reciprocal LOFO analysis showed asymmetric and range-dependent transfer between the two observed culture trajectories. KNN transferred reasonably within the overlapping density range, but its performance decreased when the held-out trajectory exceeded the maximum density represented in the training flask. These results provide a stricter assessment than the record-level split while remaining insufficient to establish generalization across independently cultivated batches.

### 3.4. Edge Deployment, Cost, and Portability

To assess whether the machine-vision pipeline could operate on portable hardware, the saved KNN pipeline was deployed locally on a Raspberry Pi 4 Model B. The saved pipeline occupied 135.90 kB. After system boot and model preload, flask loading and shield closure required approximately 13 s, image acquisition required 5 s, on-device model processing required 6 s, and shield opening and flask unloading required 13 s. These stages resulted in a ready-state load-to-unload cycle of approximately 37 s (Table 6). A complete power-on-to-shutdown test session required approximately 5 min because it additionally included system startup, model loading, and shutdown. Within the 6 s model-processing interval, image segmentation required 0.81 s, feature extraction 0.29 s, and KNN prediction 3.53 s, giving a profiled subtotal of 4.63 s. Within the 4.63 s profiled subtotal, KNN prediction accounted for 76.2%, while segmentation and feature extraction accounted for 17.5% and 6.3%, respectively. The difference between the 4.63 s profiled subtotal and the approximately 6 s total processing interval reflects additional software overhead, such as file I/O, initialization within the processing routine, and display updates.

The additional edge-device check used 10 measurements separate from the complete 240-record two-flask trajectory dataset. The saved pipeline was applied on the Raspberry Pi without refitting and produced an $R^{2}$ of 0.94. This result indicates preliminary consistency between the desktop-calibrated workflow and its local Raspberry Pi execution. Because the sample size was small and the samples were not sufficient to represent independent cultivation batches or the full operating domain, this result should be interpreted as device-level feasibility evidence rather than external validation. The reference densities of the 10 samples ranged from 20 to 80 × $10^{6}$ cells mL^−1^.

Short-term instrument repeatability was additionally evaluated using 10 consecutive RGB images acquired from an undisturbed culture flask with a reference density of approximately 45 × $10^{6}$ cells mL^−1^. The flask remained at the fixed imaging position throughout the experiment, and the camera, illumination, and acquisition settings were unchanged. The predicted densities had a mean ± SD of 45.0 ± 0.7 × $10^{6}$ cells mL^−1^, corresponding to a coefficient of variation of 1.6%. This result indicates low short-term variation in continuous image acquisition and inference under a fixed configuration. It does not quantify variability caused by flask unloading, mechanical transport, flask repositioning, flask replacement, or changes in optical conditions. Repeatability across the full calibration range therefore remains to be evaluated. The integrated hardware components had an approximate combined purchase cost of CNY 1700 (USD 254) at the time of assembly (Table 7). For international comparison, the USD equivalent was calculated using the U.S. Federal Reserve exchange rate of USD 1 = CNY 6.7060 for 16 September 2026. The USD equivalent is provided for reference; the cost estimate remains based on the original CNY purchase prices. This hardware-only estimate was calculated by summing the purchase prices of the camera, Raspberry Pi, LED light source, stepper motors, linear guide rail, PVC enclosure, display, and other electronic and assembly components. The estimate represents the acquisition cost of the assembled prototype and is dependent on the supplier, purchase date, and local market conditions. It excludes the microscope, hemocytometer, culture medium, computer used for model development, and general laboratory infrastructure.

**Figure 8:**
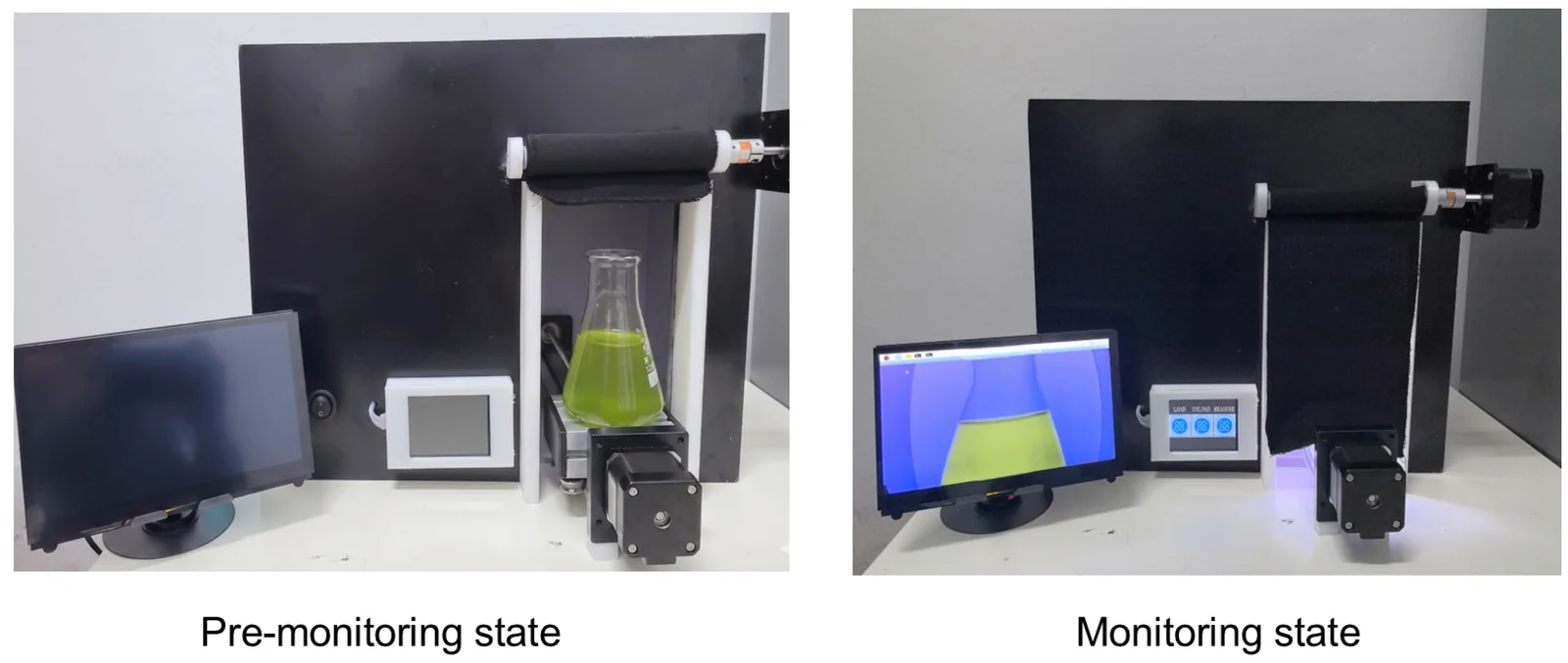
Operating states of the assembled LPMVS during the edge-device assessment. The left image shows the system before measurement, and the right image shows the culture flask positioned inside the light-isolated imaging chamber during measurement.

The assembled prototype occupied an envelope within 30 × 30 × 20 cm and weighed less than 3 kg. In this study, portability refers to a self-contained benchtop system that can be moved within a laboratory and operated without an external computer during routine measurement. Its LCD provided local control and density display without an external computer; the separate monitor visible in Figure 1 and Figure 8 only mirrored the acquired RGB image and was not required for operation. Figure 8 documents the device before monitoring and during acquisition.

The LPMVS operated locally on a Raspberry Pi with a 37 s ready-state load-to-unload cycle and approximately 6 s of model processing. Its approximate hardware cost was CNY 1700 (USD 254), and the assembled device occupied a 30 × 30 × 20 cm envelope with a mass below 3 kg. The 10-sample edge-device check and the fixed-position repeatability experiment support prototype-level operational feasibility, but they do not establish independent-batch accuracy, full-range repeatability, or cross-device transferability.

## 4. Discussion

### 4.1. Comparison with Existing Imaging Methods

The comparison in Table 1 places the present system within a broader optical-monitoring landscape rather than treating all imaging methods as equivalent. Reviews of microalgal bioprocess monitoring describe optical, fluorescence, and related approaches with different sampling and instrumentation requirements [12]. Chemical assays can provide quantitative biomass-related references [13], and fluorescence methods have been used for *Dunaliella* sp. concentration and integrity monitoring or for in-situ algal concentration estimation [14,15]. Microscopy and flow-imaging methods detect individual cells or particles and can preserve morphological information, but they require sampling, suitable dilution or a flow path, magnification, and reliable focus [29,30,35]. By contrast, bulk RGB methods measure the integrated optical response of the culture and can support non-destructive repeated monitoring after calibration [16,21,22]. The present system follows this bulk-imaging principle: it images the culture through the flask and uses a mask-derived effective liquid ROI rather than individual-cell segmentation.

Several low-cost optical platforms have demonstrated related monitoring objectives. A low-cost RGB sensor was designed for online concentration measurement in a photobioreactor [16], and a low-cost optical sensor was used to monitor and control biomass concentration during cultivation [17]. Low-cost visible–near-infrared modeling has extended estimation to density and mass [18], while another optical device has demonstrated real-time concentration measurement [19]. These studies support the feasibility of compact optical calibration, but their sensing configurations, calibration targets, reference procedures, and validation designs differ from the present flask-through RGB workflow. Their results therefore provide contextual evidence rather than directly rankable benchmarks.

Image-based studies likewise differ in the analyzed region and information content. Multivariate image analysis has been applied to microscale photobioreactors [20]; hue, saturation, and intensity features have been used for low-cost biomass quantification [21]; and RGB image processing has provided a non-invasive alternative under controlled imaging conditions [22]. Smartphone imaging [27] and modular RGB systems with real-time neural inference [28] emphasize accessibility and embedded computation, whereas computer-vision microscopy and flow-imaging microscopy retain cell- or particle-level information [29,30]. Spectral, hyperspectral, near-infrared, and quantum-dot imaging can provide richer spectral or edge information [23,24,25,26], but generally involve more specialized acquisition or processing. The LPMVS occupies a simpler bulk-RGB position: it adds automated flask handling, a mask-derived whole-liquid-region analysis, and local inference while remaining restricted to the tested optical and biological domain.

Relative to the fixed 50 × 50-pixel crop used in the camera system of Nguyen et al. [33], the mask-assisted ROI retains a larger valid liquid region while removing most dark background, flask-wall, and reflection pixels. Relative to microscope computer vision [29], routine use does not require analytical dilution, fixation, a hemocytometer, or microscopy after calibration. The dark box, diffuse LED, fixed camera position, motorized loading, and automatic shielding reduce variation in ambient illumination, viewing geometry, flask placement, and background composition. These controls improve feature comparability under the tested configuration but do not eliminate biological or cross-device sources of variation. Finally, Raspberry Pi inference and LCD display avoid dependence on an external computer during measurement.

For model calibration, prior camera-based studies have compared Gaussian-process regression [31], least-squares and Gaussian-process models [32], LASSO with image-texture features [33], and convolutional-neural-network regression [34]; deep-learning comparisons have also focused on cell counting from microscope images [35]. The internal comparison-set MAPE was 6.41%, which is numerically lower than the 8.6% average prediction error reported for the Gaussian-process system [31]. However, this comparison is descriptive only because the studies used different organisms, density ranges, reference methods, sample sizes, validation designs, and error definitions. MAPE expresses the typical error relative to reference density, whereas RMSE and MAE retain the physical unit of cell density and show the practical magnitude of prediction deviations. The lower RMSE, MAE, and MAPE of KNN relative to RF and HGB support its selection among the three evaluated regressors, while its bias near zero suggests that overprediction and underprediction were largely balanced. These results were obtained from a measurement-record split in which observations from the same flask could occur in both the training and comparison subsets; they therefore characterize record-level internal consistency. Direct numerical ranking across studies is not justified, and the present results do not establish KNN as universally superior to LASSO, Gaussian processes, or deep learning, nor do they prove independent-culture generalization.

The selected hue-, channel-, and vegetation-index descriptors indicate that the calibration was dominated by visible appearance under the tested conditions. Bootstrap resampling showed recurrent selection of *VARI*, *H_q10*, *G_iqr*, *H_q75*, *gray_skew*, and related color- or intensity-distribution descriptors in both flask-specific trajectories. This recurrence was not limited to one random training partition. However, selection frequency does not measure the independent predictive contribution of a feature group. A dedicated color-versus-texture ablation analysis would be required to quantify the incremental value of color and texture descriptors.

### 4.2. Practical Deployment and Applications

The main practical advantage is non-destructive operation after calibration. During routine measurement, the flask can be imaged and returned without removing culture for each density estimate. The system was assembled from commercially available low-cost components, automates flask positioning and light shielding, and displays density locally without an external computer. After boot and model preload, the ready-state load-to-unload cycle required approximately 37 s, including approximately 6 s of local model processing.

The component-level bill of materials, measured device envelope and mass, local display, and demonstrated edge workflow support the claims of low cost, benchtop portability, local inference, and non-destructive operation at the prototype level. The hardware acquisition cost was approximately CNY 1700 (USD 254), the device occupied a 30 × 30 × 20 cm envelope, and its mass was below 3 kg. These properties describe the assembled prototype and should not be interpreted as a standardized commercial cost or a guarantee of portability across laboratory environments.

These properties make the LPMVS most relevant to repeated monitoring of laboratory cultures maintained under a stable protocol. When periodic reference checks confirm that the calibration remains applicable, sequential density estimates may help reconstruct density trajectories and identify when confirmatory measurements are needed. This use case is consistent with earlier low-cost optical biomass-control systems [17] and recent real-time machine-learning frameworks for algal monitoring [40], while the Raman platform reported by Wieser et al. [11] illustrates a more information-rich process-integrated alternative. Density alone, however, does not determine growth phase, physiological state, product quality, or optimal harvest time. The present study did not validate growth-phase classification or harvest decisions. Such applications require complete cultivation-cycle studies that jointly measure density, physiological variables, pigment composition, and product-quality indicators.

Because the model relies substantially on color and intensity features, separately assembled devices should not be assumed to share the same calibration without verification. Differences in camera response, LED spectrum, exposure, white balance, diffuser properties, and flask geometry may require device-level calibration or calibration-transfer procedures.

### 4.3. Validation Limits and Calibration Transfer

The primary analysis contained two culture trajectories and 200 selected measurement records. Because the 8:2 split was performed at the measurement-record level, observations from the same flask could occur in both the training and internal comparison subsets. The resulting $R^{2}$ = 0.98 therefore primarily describes record-level internal consistency under the tested acquisition conditions. Reciprocal LOFO analysis using the complete 120-record trajectory from each flask provided a stricter assessment of between-flask transfer. Transfer was conditional on density-range overlap: performance was retained within the overlapping calibration domain, whereas the Flask B-trained/Flask A-tested direction showed systematic underestimation above 65.25 × $10^{6}$ cells mL^−1^. The LOFO results indicate limited extrapolation capability and should not be interpreted as complete external validation, because the two flasks were cultivated in parallel under the same protocol and optical configuration. The observed upper boundary of 100 × $10^{6}$ cells mL^−1^ should therefore be treated as a development-data boundary rather than a validated operating limit. In particular, the 90–100 × $10^{6}$ cells mL^−1^ interval contained only two primary-analysis training observations and no internal comparison observations.

The predominantly color-based calibration introduces an identifiability limitation. The LPMVS does not directly count individual cells; it estimates density from the integrated optical response of the culture. Similar cell densities may therefore produce different RGB signals when pigmentation, physiological state, nutrient status, salinity, light history, cell properties, scattering, medium composition, or optical path conditions differ. This concern is consistent with studies showing that salinity, nutrient deprivation, and related stressors alter carotenoid accumulation and the visible state of *Dunaliella* sp. cultures [7,8,9]. It is also consistent with prior image-based biomass studies that treat color calibration as condition dependent [21,22]. The most immediate determinants of the measured RGB signal are cell concentration, pigmentation, light scattering, medium composition and optical properties, illumination, and acquisition geometry. Temperature, pH, salinity, and dissolved oxygen may influence the culture physiologically, but they should not all be interpreted as direct determinants of pixel values. Cultures that shift from the visibly green state, including orange stress responses associated with altered pigment composition, fall outside the demonstrated calibration domain. The current model should therefore not be used for such cultures without additional reference-paired calibration.

Lighting and camera–flask geometry were controlled rather than experimentally varied. The present data therefore do not quantify robustness to illumination drift, flask replacement, changed fill volume, camera repositioning, altered diffuser properties, or differences between separately assembled devices. Similar low-cost optical and image-based systems also rely on repeatable acquisition and a defined optical setup [16,17,21,22]. These changes may modify the optical path, vessel boundaries, reflections, and extracted color features. The current maximum-IoU mask-selection rule is relative and lacks an experimentally validated absolute acceptance threshold. In the present prototype, the highest-IoU reference mask was selected for each image, but images with low maximum IoU were not automatically rejected. An operational system should therefore include a validated image-quality or IoU-based rejection rule that prompts repositioning or repeat acquisition when the input is outside the calibrated configuration.

Because the model relies heavily on color and intensity descriptors, calibration transfer across separately assembled devices cannot be assumed. A new device or a modified optical configuration should undergo reference-paired verification across the supported density range. If systematic disagreement is observed, device-level recalibration, color normalization, or calibration-transfer procedures will be required.

Short-term instrument repeatability was evaluated by acquiring 10 consecutive images from an undisturbed flask at approximately 45 × $10^{6}$ cells mL^−1^, without unloading, repositioning, or changing the acquisition settings. The resulting CV of 1.6% indicates limited short-term variation in continuous image acquisition and inference under a fixed configuration. It does not quantify variability introduced by flask unloading, mechanical transport, repositioning, flask replacement, or density changes. Repeatability across the full calibration range therefore remains to be established.

### 4.4. Future Development

A future multisensor extension could combine standardized flask images with turbidity, optical-density, spectral, or environmental sensors. Such combinations build on prior optical-monitoring frameworks [12,17], fluorescence-based concentration or integrity measurements [14,15], and spectral or near-infrared imaging studies [18,23,24,25]. These measurements may help distinguish density-related optical changes from variation caused by pigmentation, medium composition, and other physiological or optical factors. Their added value should be tested through direct comparisons between RGB-only and multisensor models rather than assumed from sensor availability.

Near-term validation should prioritize independently cultivated batches, multiple separately assembled devices, broader and better-balanced density coverage, and repeated-imaging experiments that include both fixed-position acquisition and flask repositioning. Feature selection and calibration transfer should also be evaluated across cultivation batches and devices. Additional experiments should examine the effects of flask replacement, nominal flask capacity, working volume, fill height, and medium preparation variability on the extracted image features and density predictions. Only after transferability and repeatability are established should downstream tasks such as growth-phase estimation or harvest forecasting be evaluated.

## 5. Conclusions

This study developed a low-cost portable machine-vision system (LPMVS) for non-destructive density estimation of visibly green *Dunaliella* sp. cultures under controlled laboratory conditions. K-nearest neighbors (KNN) performed best among the three evaluated regressors in the record-level internal comparison ($R^{2}$ = 0.98; MAPE = 6.41%). Reciprocal leave-one-flask-out analysis yielded full-trajectory $R^{2}$ values of 0.914 for Flask A → B and 0.803 for Flask B → A. Transfer was stronger within the overlapping density range, whereas densities above the training range were systematically underestimated. These results support feasibility within the tested calibration domain but do not establish generalization to independent cultivation batches.

The self-contained prototype cost approximately CNY 1700 (USD 254), occupied a 30 × 30 × 20 cm envelope, and weighed less than 3 kg. It completed a ready-state load-to-unload cycle in approximately 37 s, including approximately 6 s of model processing. The edge-device check and fixed-position repeatability assessment provided preliminary evidence of operational feasibility. The principal contribution is the integration of controlled optical acquisition, liquid-region analysis, and local edge inference. Wider use requires independent-batch validation, broader density coverage, assessment of flask-repositioning variability, and verification of calibration transfer across devices.

## Figures and Tables

**Table 1:** Representative image-based strategies for microalgal density or concentration measurement and their deployment-relevant characteristics. Entries are qualitative summaries based on the cited reports; image regions, illumination and geometry, cost, and deployment characteristics were not extracted under a common experimental protocol and should not be interpreted as directly comparable measurements.

Approach and Source	Image Basis	Analyzed Region	Illumination and Geometry	Deployment-Relevant Trade-Off
Bulk color analysis [16,21,22]	Red–green–blue (RGB)/hue–saturation–intensity (HSI) culture images	Culture appearance or color statistics	Defined acquisition configuration reported by each source	Low-cost and non-destructive; normally calibrated for a defined optical setup
Camera regression [31,32,33,34]	RGB image of a transparent sample container	System-specific image region; a fixed 50 × 50-pixel crop in [33]	Source-specific acquisition geometry	Simple image input, but calibration and analyzed-region choices remain source-specific
Hemocytometer computer vision [29]	40× microscope images	Entire microscope field; individual-cell detection	Microscope-based acquisition; focus and illumination affect detection	Direct cell enumeration, but sampling, dilution, a chamber, and microscopy remain necessary
Flow-imaging microscopy [30]	Flow-cell micrographs	Individual particles or cells	Controlled flow-imaging optics	Rich cell-level information, with additional flow and microscopy hardware
Smartphone/modular imaging [27,28]	Platform-specific RGB images	Device-specific field or region of interest (ROI)	Performance depends on acquisition configuration	Accessible hardware; computational and training requirements depend on the model
This study	RGB image through a culture flask	Mask-derived effective liquid ROI	Dark box, diffuse LED, fixed camera–flask geometry	Non-destructive automated handling with Raspberry Pi prediction and LCD display

**Table 2:** Optimal hyperparameters of the k-nearest neighbors (KNN), random forest (RF), and histogram-based gradient boosting (HGB) models for the primary record-level comparison. Hyperparameter optimization was repeated using only the training flask in each LOFO direction.

Model	Hyperparameter	Selected Value
KNN	Scaler	StandardScaler
	n_neighbors	17
	*p*	2
	Weights	Uniform
	Leaf size	20
RF	n_estimators	1200
	Maximum depth	10
	Minimum samples split	2
	Minimum samples leaf	1
	Maximum features	log2
	Bootstrap	True
HGB	Learning rate	0.02
	Maximum iterations	300
	Maximum depth	5
	Maximum leaf nodes	31
	Minimum samples leaf	20
	L2 regularization	0.00

**Table 3:** Reference-density coverage of the complete culture trajectories used for flask-specific bootstrap and reciprocal leave-one-flask-out (LOFO) analyses. Density values are expressed in $10^{6}$ cells mL^−1^.

Culture Trajectory	*n*	Density Range ($\times 10^{6}$ Cells mL^−1^)	≤65.25 ($\times 10^{6}$ Cells mL^−1^)	>65.25 ($\times 10^{6}$ Cells mL^−1^)
Flask A	120	2.5–100	79	41
Flask B	120	2.5–65.25	120	0

**Table 4:** Record-level internal comparison performance of the optimized KNN, RF, and HGB models under the 8:2 measurement-record split. Point estimates are shown with 95% bootstrap confidence intervals in brackets. RMSE, MAE, and bias are expressed in $10^{6}$ cells mL^−1^; bias was calculated as predicted minus reference density. Because records from the same culture flask could occur in both the training and comparison subsets, these results quantify record-level internal consistency rather than independent-culture generalization.

Model	$R^{2}$	RMSE	MAE	MAPE (%)	Bias
KNN	0.98 [0.97, 0.99]	2.84 [2.20, 3.45]	2.16 [1.61, 2.76]	6.41 [4.62, 8.38]	−0.42 [−1.29, 0.43]
RF	0.97 [0.94, 0.98]	3.87 [2.93, 4.70]	2.96 [2.20, 3.76]	8.74 [6.22, 11.54]	−1.10 [−2.23, 0.03]
HGB	0.92 [0.87, 0.96]	5.88 [4.37, 7.26]	4.34 [3.17, 5.61]	12.91 [8.89, 17.35]	−1.77 [−3.49, −0.05]

**Table 5:** Reciprocal leave-one-flask-out transfer performance obtained by independently retraining the complete pipeline in each direction. Density values and RMSE, MAE, and bias are expressed in $10^{6}$ cells mL^−1^. Bias was calculated as predicted minus reference density. The full-range results include all 120 records in the held-out flask. The common-range results for the Flask B → A direction were restricted to reference densities ≤$65.25\times 10^{6}$ cells mL^−1^, corresponding to the maximum density represented in the Flask B training trajectory. The 41 Flask A records above this boundary were treated as extrapolation observations.

Training → Test	Test Domain	*n*	Ref. Density Range	Model	$R^{2}$	RMSE	MAE	Bias
A → B	Full held-out trajectory	120	2.5–65.25	**KNN**	**0.914**	**4.109**	**3.470**	−0.157
A → B	Full held-out trajectory	120	2.5–65.25	RF	0.866	5.139	4.874	+1.069
A → B	Full held-out trajectory	120	2.5–65.25	HGB	0.854	5.368	4.534	−1.851
B → A	Full held-out trajectory	120	2.5–100	**KNN**	**0.803**	**13.453**	**9.384**	−5.426
B → A	Full held-out trajectory	120	2.5–100	RF	0.668	17.454	12.844	−7.129
B → A	Full held-out trajectory	120	2.5–100	HGB	0.734	15.630	11.590	−6.790
B → A	Overlapping range	79	2.5–65.25	**KNN**	**0.936**	**4.669**	**4.095**	+1.917
B → A	Overlapping range	79	2.5–65.25	RF	0.885	6.256	5.207	+3.474
B → A	Overlapping range	79	2.5–65.25	HGB	0.920	5.226	4.701	+2.589

Bold values indicate the best performance within each evaluation setting (highest $R^{2}$, lowest RMSE and MAE, and bias closest to zero); bold model names identify the corresponding model.

**Table 6:** Runtime profile and operational timing of the Raspberry Pi-based LPMVS workflow. The 37 s ready-state cycle began after system boot and model preload and included flask loading, shield closure, image acquisition, model processing, shield opening, and flask unloading. The approximately 5 min full session additionally included Raspberry Pi startup, model loading, and shutdown. Processing-stage shares were calculated only from the 4.63 s subtotal of the three profiled stages and therefore do not represent the full 6 s model-processing interval.

Workflow Component	Time (s)	Share of Profiled Subtotal (%)
Flask loading and shield closure	≈13	–
Image acquisition	5.00	–
Image segmentation	0.81	17.5
Feature extraction	0.29	6.3
KNN prediction	3.53	76.2
Profiled-stage subtotal	4.63	100.0
Complete model-processing interval	6.00	–
Shield opening and flask unloading	≈13	–
Ready-state load-to-unload cycle	≈37	–
Power-on-to-shutdown test session	≈300	–

**Table 7:** Bill of materials and approximate acquisition cost of the low-cost portable machine-vision system (LPMVS). Costs are approximate purchase prices in Chinese yuan at the time of assembly. The total represents the integrated prototype hardware cost and excludes the microscope, hemocytometer, culture medium, model-development computer, and general laboratory infrastructure.

Component	Quantity	Approx. Cost (CNY)
IMX477 camera	1	472
Raspberry Pi	1	485
LED light source	1	33
Stepper motors	2	50
Linear guide rail	1	197
PVC enclosure	1	180
Display screen	1	160
Other electronic and assembly components	Several	120
**Total**	–	**CNY 1700 (USD 254)**

Note: The total is approximately CNY 1700 (USD 254), with the USD equivalent calculated using the U.S. Federal Reserve exchange rate of USD 1 = CNY 6.7060 for 16 September 2026. The USD equivalent is provided for reference; the cost estimate remains based on the original CNY purchase prices.

## Data Availability

The image data, reference-density measurements, feature-extraction scripts, model-training code, and Raspberry Pi deployment code are available from the corresponding author upon reasonable request.
